# The Relationship between Polyamines and Hormones in the Regulation of Wheat Grain Filling

**DOI:** 10.1371/journal.pone.0078196

**Published:** 2013-10-29

**Authors:** Yang Liu, Dandan Gu, Wei Wu, Xiaoxia Wen, Yuncheng Liao

**Affiliations:** 1 College of Agronomy, Northwest A&F University, Yangling, Shaanxi, China; 2 College of Plant Protection, Northwest A&F University, Yangling, Shaanxi, China; National Taiwan University, Taiwan

## Abstract

The grain weight of wheat is strongly influenced by filling. Polyamines (PA) are involved in regulating plant growth. However, the effects of PA on wheat grain filling and its mechanism of action are unclear. The objective of the present study was to investigate the relationship between PAs and hormones in the regulation of wheat grain filling. Three PAs, spermidine (Spd), spermine (Spm), and putrescine (Put), were exogenously applied, and the grain filling characteristics and changes in endogenous PA and hormones, i.e., indole-3-acetic acid (IAA), zeatin (Z) + zeatin riboside (ZR), abscisic acid (ABA), ethylene (ETH) and gibberellin 1+4 (GAs), were quantified during wheat grain filling. Exogenous applications of Spd and Spm significantly increased the grain filling rate and weight, but exogenous Put had no significant effects on these measures. Exogenous Spd and Spm significantly increased the endogenous Spd, Spm, Z+ZR, ABA, and IAA contents and significantly decreased ETH evolution in grains. The endogenous Spd, Spm and Z+ZR contents were positively and significantly correlated with the grain filling rate and weight of wheat, and the endogenous ETH evolution was negatively and significantly correlated with the wheat grain filling rate and weight. Based upon these results, we concluded that PAs were involved in the balance of hormones that regulated the grain filling of wheat.

## Introduction

The yield potential of wheat (*Triticum aestivum* L.) is divided into the following three major components: the panicle number per plant, the grain number per panicle, and the grain weight. Grain filling, the final stage of cereal growth, determines the grain weight [1]. Modern high yield crop production systems require high yield outputs, and for this reason, improved grain filling has become more important than ever [2], [3].

Polyamines (PA) are organic polycations, which are low molecular weight nitrogen-containing compounds [4]. They have been described as endogenous plant growth regulators or intracellular messengers that regulate plant growth, development, and responses to abiotic stresses [5], [6], [7], [8]. In addition, PA was thought to be involved in the regulation of grain development. The PA content of normal kernels was significantly higher than that of aborting maize kernels (*Zea mays* L.), and the PA content was positively correlated with the endosperm nuclei number [9]. Yang et al. [10] found that higher levels of spermidine (Spd, one PA) and spermine (Spm, another PA) promote grain filling and increase the grain weight of rice (*Oryza sativa* L.); however, putrescine (Put, another PAs) had the opposite effect. Tan et al. [11] suggested that low concentrations of Spd and Spm and low Spd/Put and Spm/Put ratios may be important physiological causes for the low grain filling rate and the low grain weight of inferior spikelets in super rice. However, the effect of PA on the regulation of wheat grain filling and its mechanism remain unclear.

Plant hormones play an important role in regulating grain development. High levels of cytokinins (CTK) are generally found in the developing grains of cereals, peas, and beans [12], [13], [14], [15], [16]. CTKs are thought to be involved in cell division during seed development [16]. In wheat, superior grains had a higher abscisic acid (ABA) content and a lower ethylene (ETH) content compared with inferior grains, and the ratio of ABA/ETH was positively and significantly correlated with the grain filling rate [17]. In addition, the grain filling rate was positively correlated with the indole-3-acetic acid (IAA) content of rice grains [18]. High gibberellin 1 (GA_1_) and GA_19_ levels were found in the large panicles of rice immediately before and at anthesis [19].

PA and ETH reportedly share the same S-adenosylmethionine biosynthetic precursor, and increasing PA biosynthesis has a notable effect on ETH synthesis rates [9]. Exogenous PA represses ETH synthesis in oat (*Avena sativa* L.) leaves [20] and rice panicles [10]. In addition, exogenous ABA increased the Put content in chickpeas (*Cicer arietinum* L.) [21]. This reduced endogenous ABA content led to a decrease in the PA levels in maize [22]. These studies provided clear evidence that there is a close relationship between PA and hormones in the regulation of plant growth. However, little is known about the relationship between PA and hormones in the regulation of wheat grain filling.

In the present study, two widely grown winter wheat cultivars from northern China were selected and treated with external Spm, Spd and Put, and then the grain filling characteristics and changes in PA (Spm, Spd and Put), IAA, zeatin (Z) + zeatin riboside (ZR), ABA, ETH, and GA_1+4_ in the grains were measured during wheat grain filling. The objective of the present study was to investigate the relationship of PA and hormones with the regulation of wheat grain filling and to determine whether the grain filling of wheat can be regulated by manipulating the PA levels.

## Materials and Methods

### Study site description

This study was conducted from 2011 to 2013 at the Crop Specimen Farm in Northwest A&F University, Shaanxi Province, northwestern China. The latitude and longitude of the experimental station are 34°22′N and 108°26′E, respectively. The annual mean precipitation of the experimental station is 550 mm. The annual mean maximum and minimum air temperatures at the site are 42°C and −19.4°C, respectively, and the annual mean temperature is 12.9°C. The total yearly sunshine duration is 2196 h, and the frost-free period is 220 days. The precipitation was 255 mm and 213 mm during the wheat growth stages during 2011–2012 and 2012–2013, respectively. The soil at the experiment farm is Eum-Orthrosols (Chinese soil taxonomy), and the mean bulk density of the soil was 1.31 g cm^−3^. The readily available N, P and K quantities were 58.27 mg kg^−1^, 19.26 mg kg^−1^, and 125.38 mg kg^−1^, respectively. The organic matter content of the 0–20 cm topsoil was 12.21 g kg^−1^, and the pH was 7.35.

### Experiment design and sampling

Two winter wheat cultivars, Zhoumai 18 and Xinong 538, were grown in the field. Seeds were sown on Oct 10, 2011 and Oct 14, 2012. The sowing density was 150 kg ha^−1^, and the row spacing was 0.20 m. Overall, 150 kg ha^−1^ urea and 150 kg ha^−1^ diammonium orthophosphate were applied at basal levels. At anthesis, 1 mmol L^−1^ Spd (S1), 1 mmol L^−1^ Spm (S2), and 2 mmol L^−1^ Put (P1) were sprayed on the wheat panicles with a sprayer. The chemicals were applied daily for five days at a rate of 5 ml per spike for each application. All of the solutions contained 0.1% (V/V) ethanol and 0.01% (V/V) Tween-20. The same volume of deionized water containing the same concentrations of ethanol and Tween-20 was applied to the control plants (CK). Each treatment had three replicates with a completely randomized block design and plot dimensions of 3 m×3 m. The Spm, Spd, and Put were purchased from Sigma-Aldrich (USA).

For each plot, 400 spikes that flowered on the same day were chosen and marked. Twenty spikes from each plot were marked and sampled at three-day intervals from anthesis to maturity. All of the grains on the spikes were removed. Grains on the middle spikelets (four to 12 spikelets from the bottom) were used for the measurement. A total of 60 to 80 sampled grains were used to measure the ETH evolution. Half of the sampled grains were frozen in liquid nitrogen for 15 min and then stored at −40°C to measure the levels of other hormones and PA. The other grains were dried at 75°C to a constant weight and weighed.

### Measurement

#### Grain filling process

The grain filling process was calculated using the Richards [23] growth equation and in accordance with Yang et al. [17] as follows: 
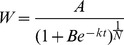
(1)The grain-filling rate (*G*) was calculated as the derivative of Eqn. 1: 
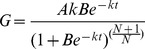
(2)[*W*, the grain weight (mg); *A*, the final grain weight (mg); *t*, time after anthesis (d); *B*, *k*, and *N*, coefficients determined by regression.]

The active grain-filling period was defined as the period when *W* was between 5% (*t*_1_) and 95% (*t*_2_) of A. Therefore, the average grain-filling rate during this period was calculated from *t*_1_ to *t*_2_ [17].

#### Free Pas

Free Spd, Spm, and Put were extracted and measured according to Yang et al. [10] and Liu et al. [24]. Briefly, the sampled grains (0.5–1.0 g) were homogenized in a pre-chilled mortar and pestle in 3–5 ml of 5% (v/v) perchloric acid (PCA). The homogenates were incubated at 5°C for 1 h and centrifuged at 25,000× *g* for 20 min, and the supernatant was then collected. PAs in the supernatant were derived with benzoyl chloride and quantified by a high-performance liquid chromatography system (P680 Pump/UVD170U UV-VIS Detector, DIONEX, USA). Exactly 20 µL of each methanol (60% v/v)-redissolved sample was injected and loaded onto a 4.6 mm by 250 mm 5-µm particle size reverse-phase (C18) column (Waters). The column temperature was 25°C, with a flow rate of 0.6 ml min^−1^. Polyamine peaks were detected at an absorbance of 254 nm.

#### Hormones

Approximately 0.5 g FW of grain was sampled, and each endogenous hormone concentration was measured. The Z+ZR, GAs (GA_1_+GA_4_), IAA and ABA were extracted and purified according to previous studies [25], [26]. The samples were ground in a mortar (on ice) with 5 ml of 80% (v/v) methanol as the extraction medium, which contained 1 mmol L^−1^ butylated hydroxytoluene (BHT) as an antioxidant. The methanolic extracts were incubated at 4°C for 4 h and centrifuged at 10,000× *g* for 15 min at the same temperature. The supernatants were passed through Chromosep C18 columns (C18 Sep-Park Cartridge, Waters Corp., Millford, MA, USA). The columns were pre-washed with 10 ml of 100% methanol and 5 ml of 80% methanol. The hormone fractions were dried with N_2_ and dissolved in 1 ml of phosphate-buffered saline (PBS) containing 0.1% (v/v) Tween 20 and 0.1% (w/v) gelatin (pH 7.5) for an enzyme-linked immunosorbent assay (ELISA).

The mouse monoclonal antigens and antibodies against Z+ZR, GAs (GA1+GA4), IAA, and ABA and the immunoglobulin G-horseradish peroxidase (IgG-HRP) used in the ELISA were manufactured by the Phytohormones Research Institute, China Agricultural University. The quantification of Z+ZR, GAs (GA_1_+GA_4_), IAA, and ABA was performed by ELISA as previously described [25], [26]. The recovery rates for IAA, Z+ZR, ABA, and GAs were 86.3±5.5%, 89.1±4.4%, 87.2±2.8%, and 79.7±6.1%, respectively.

The ethylene produced by the grains was determined according to Yang et al. [17]. Briefly, the sampled grains were placed between two sheets of moist paper for 1 h at 27°C in darkness to allow the wound-inducing ethylene production to subside. The grains were then transferred to 25-ml glass vials containing moist filter paper, and the vials were immediately sealed with airtight Suba-Seal stoppers and incubated in the dark for 8 h at 27°C. A 1-ml gas sample was withdrawn through the Suba-Seal using a gas-tight syringe, and the ethylene level was assayed with a gas chromatograph (Trace GC UItra, Thermo Fisher Scientific, USA) equipped with a Porapak Q column (0.3 cm×200 cm, 0.18–0.30 mm) and a flame ionization detector (FID). The temperatures for the injection port, column, and detector were maintained at 70, 70, and 150°C, respectively. Nitrogen was used as a carrier gas at a flow rate of 40 KPa, and hydrogen and air were used for the FID measurements at 35 and 350 ml min^−1^, respectively. The rate of ethylene evolution was expressed as a function of the unit fresh weight (FW).

### Yield and yield components

Plants (except those along the border) from a 2-m^2^ site in each plot were harvested at maturity to determine the grain yield. Yield components, e.g., the spikes per square meter, grain number per spike, and grain weight, were determined from plants harvested from a 1-m^2^ site (excluding the border plants) randomly sampled from each plot.

### Statistical analysis

The results were analyzed for variance using SPSS 16.0 for Windows. The data from each sampling set were analyzed separately. The means were tested by the least significant difference method at *P = 0.05* (LSD 0.05). The differences in grain weights, grain filling characteristics, PA levels, and hormone levels across the two study years were not significant (F<1). Therefore, the grain filling characteristic data and the PA and hormone level data from the two years were averaged.

## Results

### Yield and yield components

The external Spd, Spm, and Put applications had different effects on the grain yield of wheat. The external applications of Spd and Spm significantly increased the grain yield of wheat; however, the external Put had no significant effect (Table 1). The two cultivars showed the same trend during the two experiment stages. In addition, Table 1 showed that the external Spd and Spm significantly increased the grain weight; however, the external Spd and Spm had no significant effects on the spike number and grain number per spike.

**Table 1:** Effects of polyamine on the yield and yield components of winter wheat.

Year	Cultivar	Treatment	Spike number (×10^4^ hm^−2^)	Grain number per spike	1000-grain weight (g)	Grain yield (t hm^−2^)
2011–2012	Zhoumai 18	Spd	415.1 a	33.4 a	51.6 a	7.3 a
		Spm	414.3 a	33.5 a	51.3 a	7.3 a
		Put	414.9 a	33.1 a	48.9 b	6.7 b
		CK	415.0 a	32.8 a	49.6 b	6.8 b
	Xinong 538	Spd	452.3 a	33.9 a	41.9 a	6.7 a
		Spm	450.9 a	34.3 a	42.3 a	6.7 a
		Put	451.8 a	33.5 a	39.5 b	6.0 b
		CK	451.8 a	33.7 a	40.1 b	6.1 b
2012–2013	Zhoumai 18	Spd	406.8 a	34.5 a	52.1 a	7.3 a
		Spm	407.7 a	34.4 a	52.2 a	7.4 a
		Put	405.3 a	34.2 a	49.3 b	6.8 b
		CK	405.9 a	33.9 a	49.7 b	6.8 b
	Xinong 538	Spd	443.6 a	32.8 a	42.5 a	6.2 a
		Spm	442.4 a	33.4 a	42.6 a	6.3 a
		Put	441.8 a	32.4 a	39.5 b	5.7 b
		CK	442.3 a	32.5 a	39.8 b	5.7 b

Values within a column for the same cultivar and the same year followed by different letters are significantly different at *P* = 0.05. S1: external Spd application on panicles; S2: external application of Spm on panicles; P1: external application of Put on panicles.

### Grain filling

**Figure 1:**
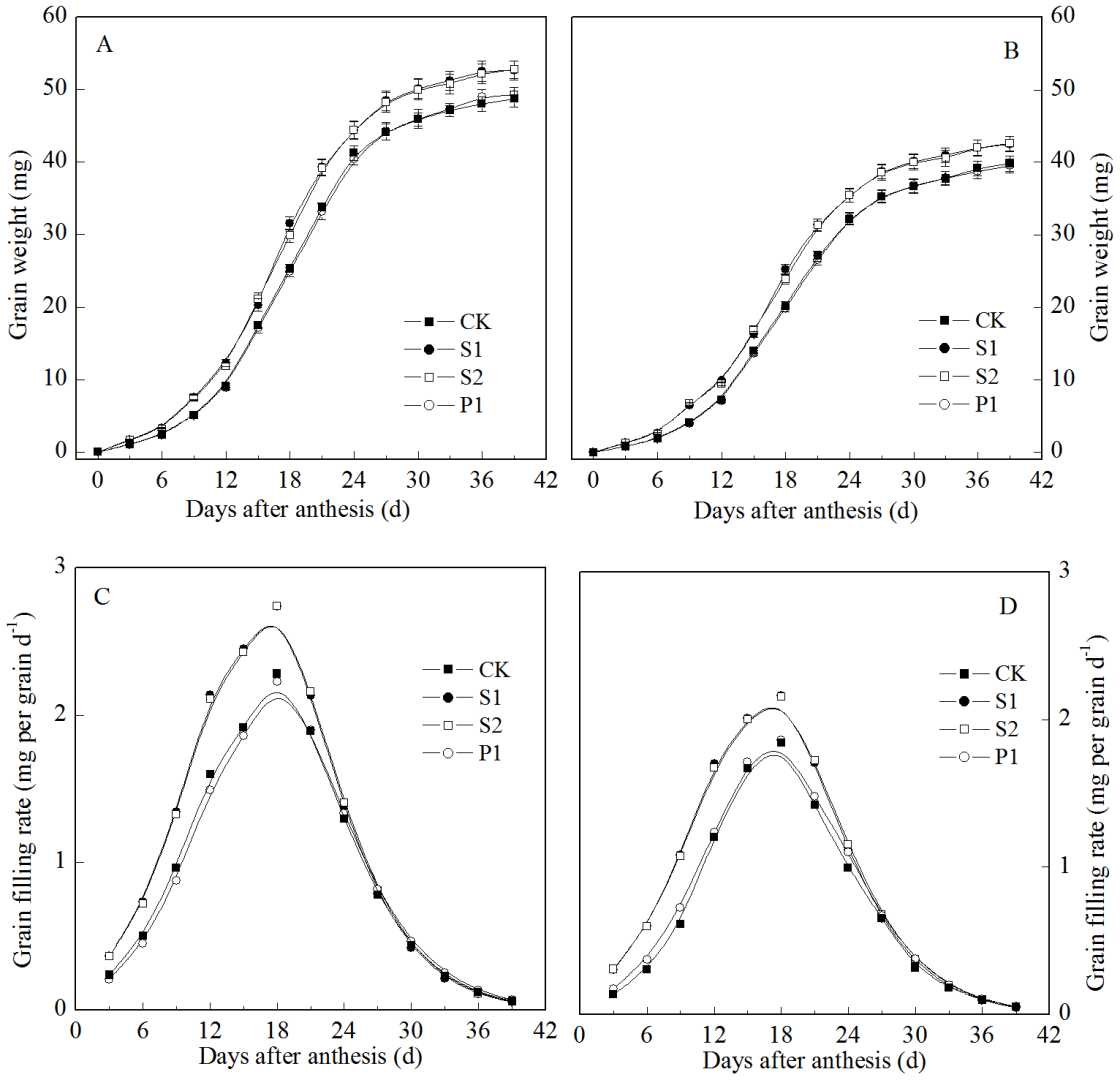
External PA effects on the grain weights (A: Zhoumai 18; B: Xinong 538) and grain filling rates (C: Zhoumai 18; D: Xinong 538) in winter wheat. S1: external application of Spd on panicles; S2: external application of Spm on panicles; P1: external application of Put on panicles; CK: external application of deionized water on panicles. Vertical bars represent ± the standard error of the mean (n = 3).

There was a significant difference in the grain weights between Zhoumai 18 and Xinong 538 (Fig. 1). The grain weight of Zhoumai 18 was significantly higher than that of Xinong 538, and Zhoumai 18 had a higher grain filling rate than Xinong 538 (Fig. 1, Table 2).

**Table 2:** Polyamine effects on the maximum grain filling rate (G_max_), mean grain filling rate (G_mean_), and maximum grain weight (W_max_) of winter wheat.

	Zhoumai 18				Xinong 538			
	S1	S2	P1	CK	S1	S2	P1	CK
G_max_ (mg per grain d^−1^)	2.74a	2.73a	2.23b	2.28b	2.16a	2.16a	1.86b	1.84b
G_mean_ (mg per grain d^−1^)	2.15a	2.15a	1.95b	1.94b	1.76a	1.77a	1.42b	1.40b
W_max_ (mg)	52.6a	52.5a	49.4b	48.5b	42.3a	42.3a	39.4b	39.8b

Values in each row for the same cultivar followed by different letters are significantly different at *P* = 0.05. S1: external application of Spd on panicles; S2: external application of Spm on panicles; P1: external application of Put on panicles.

The different PAs had different effects on wheat grain filling (Fig. 1); external applications of Spd and Spm significantly promoted grain filling. During the filling stage, the grain weights of the S1 and S2 treatments were significantly higher than that of CK on the same day. Table 2 shows that the maximum grain weights and the maximum and mean grain-filling rates for the S1 and S2 treatments were significantly higher than those of the CK treatment. In contrast, exogenous Put had no significant effect on the grain filling of wheat. There was no significant difference in the grain weights between the P1 and CK treatments during the grain filling stage. The maximum grain weight and the maximum and mean grain-filling rate of the P1 treatment also showed no significant differences compared with that of the CK treatment.

### Polyamine changes

**Figure 2:**
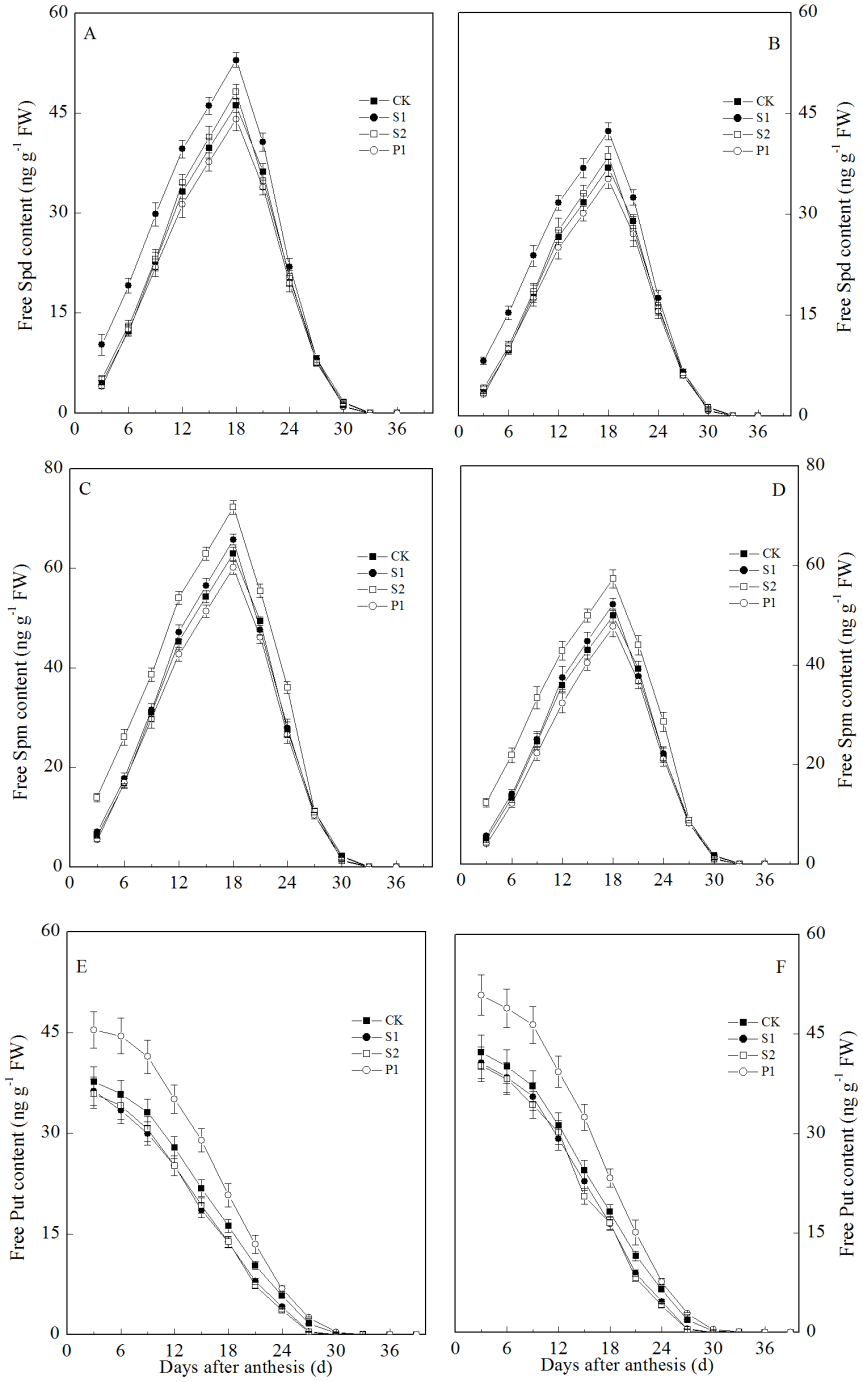
External PA effects on the Spd (A: Zhoumai 18; B: Xinong 538), Spm (C: Zhoumai 18; D: Xinong 538), and Put (E: Zhoumai 18; F: Xinong 538) contents in wheat grains. S1: external application of Spd on panicles; S2: external application of Spm on panicles; P1: external application of Put on panicles; CK: external application of deionized water on panicles. Vertical bars represent ± the standard error of the mean (n = 3).

The free Spm and Spd contents in the grains transiently increased at the early grain filling stage, reaching a maximum at 18 days after anthesis and decreasing thereafter (Fig. 2). The two cultivars showed similar trends. In contrast to these results, the free Put content of the grains decreased gradually during the grain filling stage. An external application of Spm, Spd, or Put significantly increased the Spm, Spd, or Put contents of the grain, respectively.

### Hormonal changes

**Figure 3:**
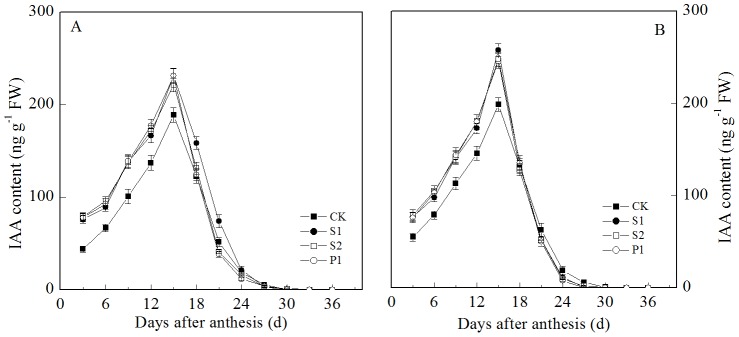
External PA effects on the IAA content (A: Zhoumai 18; B: Xinong 538) in wheat grains. S1: external application of Spd on panicles; S2: external application of Spm on panicles; P1: external application of Put on panicles; CK: external application of deionized water on panicles. Vertical bars represent ± the standard error of the mean (n = 3).

**Figure 4:**
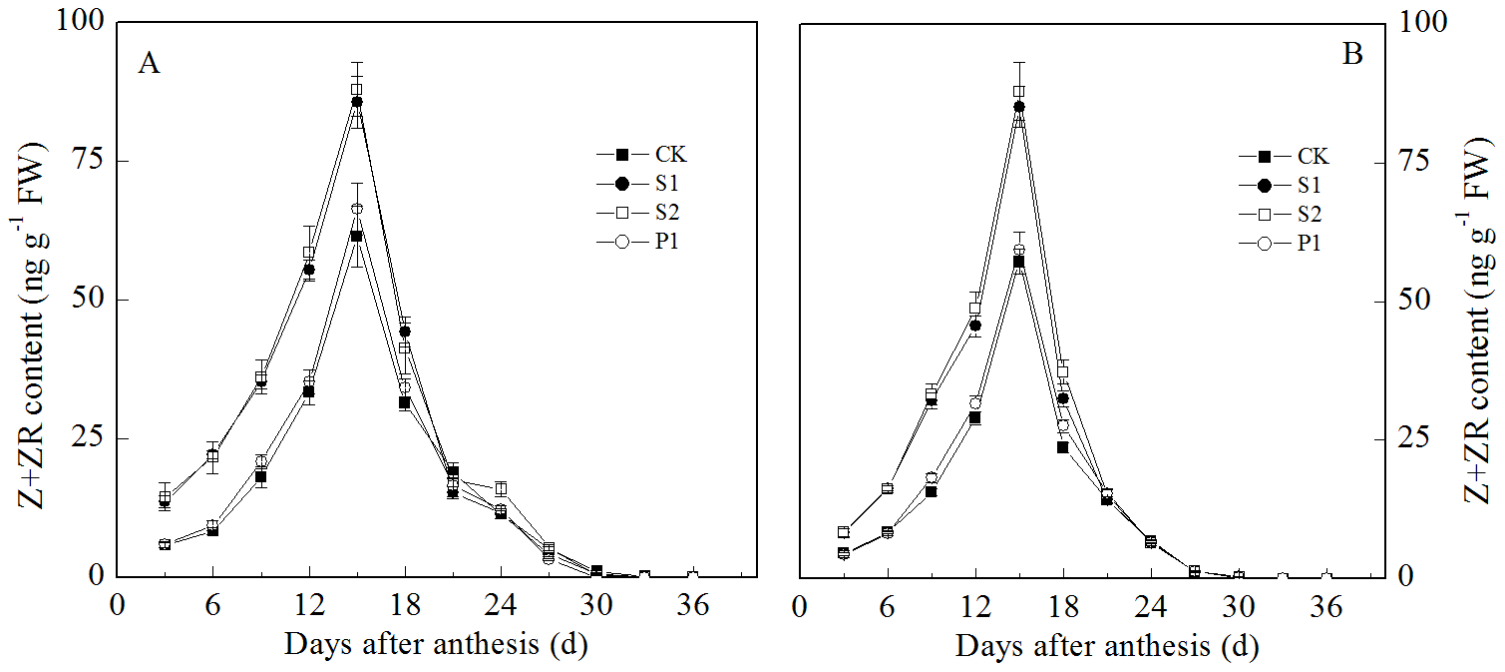
External PA effects on the Z+ZR content (A: Zhoumai 18; B: Xinong 538) in wheat grains. S1: external application of Spd on panicles; S2: external application of Spm on panicles; P1: external application of Put on panicles; CK: external application of deionized water on panicles. Vertical bars represent ± the standard error of the mean (n = 3).

The IAA and Z+ZR contents in the grains all transiently increased at the early grain filling stage, reaching a maximum at 15 days after anthesis and decreasing thereafter (Fig. 3 and 4). The two cultivars showed similar trends. The external applications of Spm, Spd, and Put all significantly increased the IAA content in the grains during the early and middle grain filling stages. The IAA contents in the grains subjected to the S1, S2, and P1 treatments were all significantly higher than those of the CK treatment at three to 15 days post-anthesis. In contrast to the IAA, the different PA levels had different effects on the grain Z+ZR contents. The external Spd and Spm significantly increased the Z+ZR contents in the grains during the early and middle grain filling stages. The Z+ZR contents in the grains treated with S1 and S2 were significantly higher than those of the CK from three to 15 d post-anthesis. However, there was no significant difference in the Z+ZR contents of grains between the P1 and CK treatments during the grain filling stage.

**Figure 5:**
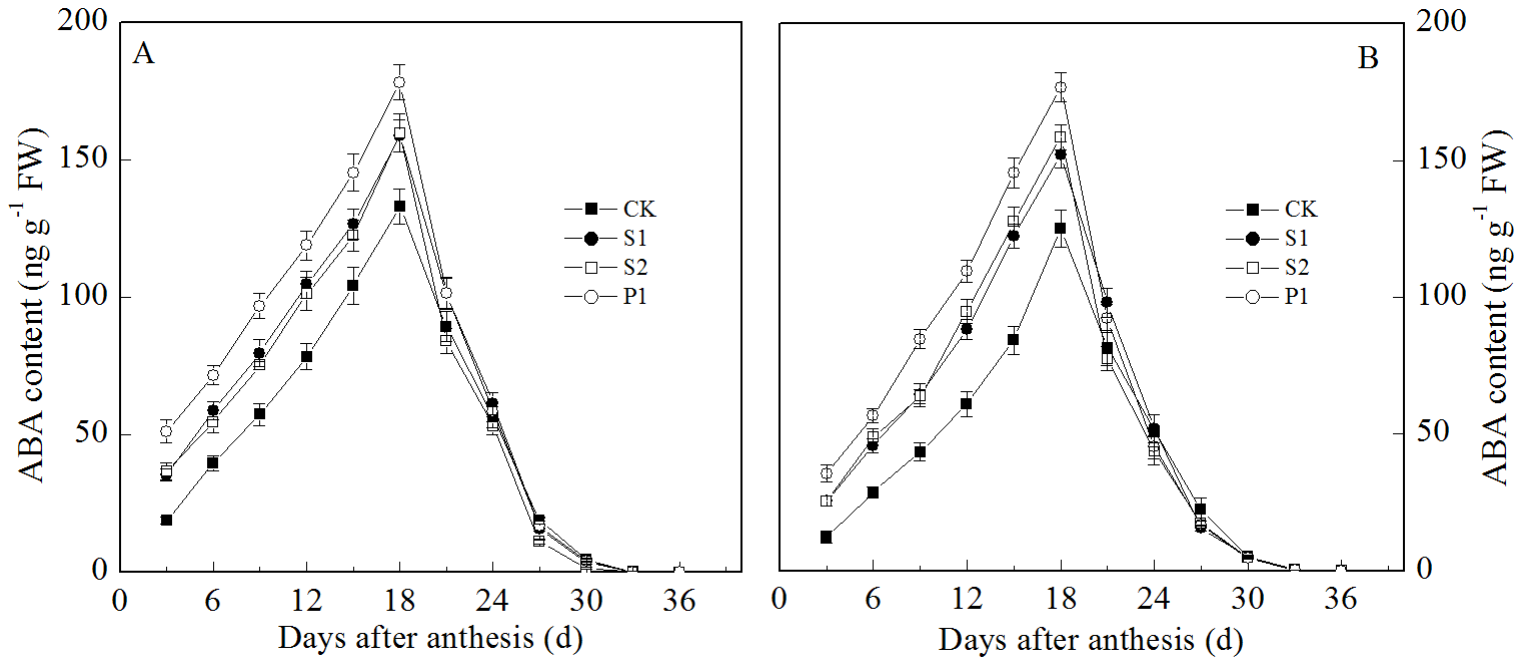
External PA effects on the ABA content (A: Zhoumai 18; B: Xinong 538) in wheat grains. S1: external application of Spd on panicles; S2: external application of Spm on panicles; P1: external application of Put on panicles; CK: external application of deionized water on panicles. Vertical bars represent ± the standard error of the mean (n = 3).

Similar to the IAA and Z+ZR contents, the ABA grain content transiently increased at the early grain filling stage and then decreased (Fig. 5). However, the ABA content in the grains reached a maximum at 18 days after anthesis for the two cultivars. External applications of Spd, Spm, and Put all significantly increased the ABA content in the wheat grains. The ABA contents in the grains from the S1, S2, and P1 treatments were all significantly higher than that of the CK treatment from three to 15 days after anthesis. In addition, the ABA content of the grains from the P1 treatment was significantly higher than those of the S1 and S2 treatments; compared with Spd and Spm, the externally applied Put had a more notable effect on the ABA content in the grains.

**Figure 6:**
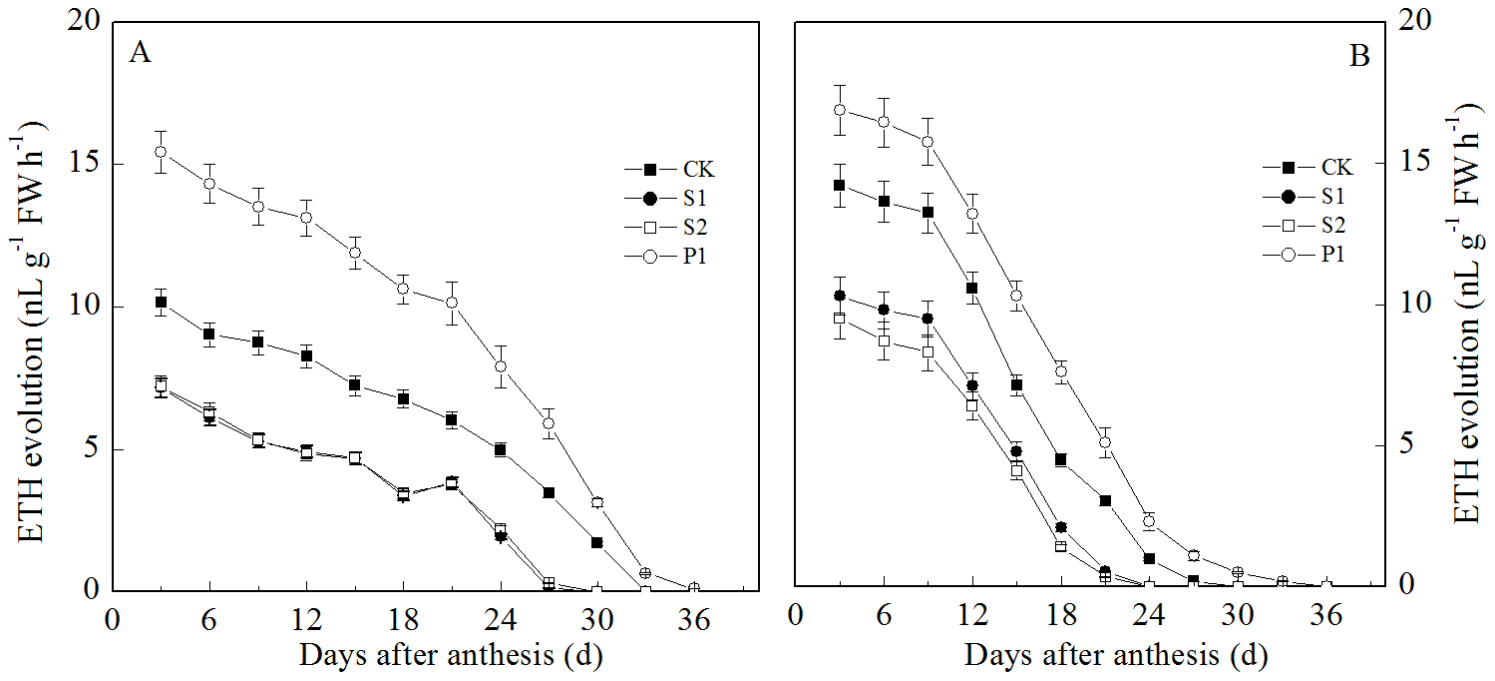
External PA effects on the ETH production rate (A: Zhoumai 18; B: Xinong 538) in wheat grains. S1: external application of Spd on panicles; S2: external application of Spm on panicles; P1: external application of Put on panicles; CK: external application of deionized water on panicles. Vertical bars represent ± the standard error of the mean (n = 3).

**Figure 7:**
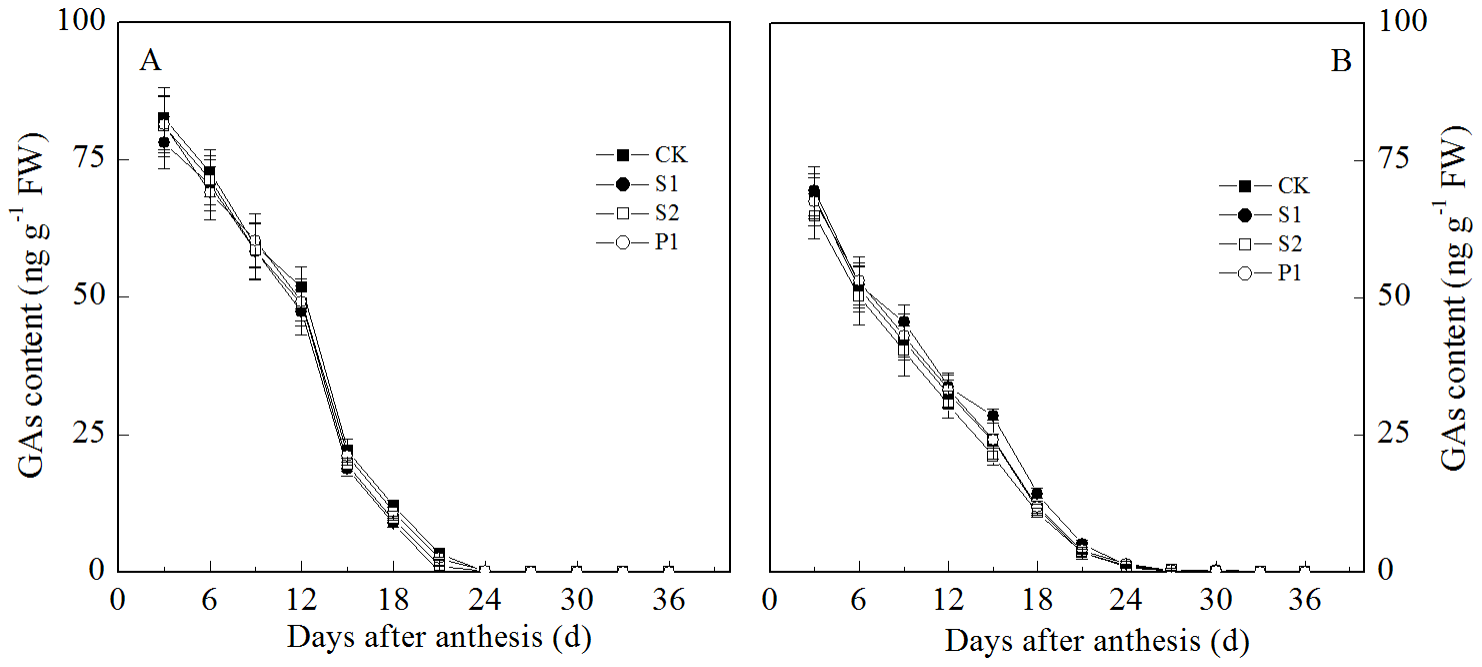
External PA effects on the GA content (A: Zhoumai 18; B: Xinong 538) in wheat grains. S1: external application of Spd on panicles; S2: external application of Spm on panicles; P1: external application of Put on panicles; CK: external application of deionized water on panicles. Vertical bars represent ± the standard error of the mean (n = 3).

During the grain filling stage, the ETH and GA contents in the grains gradually decreased (Fig. 6 and 7). The external PA, Spd, Spm, and Put all significantly affected the ETH content in the grains. However, the different PAs had different effects on the grain filling of wheat. The external Spd and Spm significantly decreased the ETH content in the wheat grains, whereas the external Put significantly increased the ETH content in the grains during the grain filling stage. In contrast to ETH, the external Spd, Spm, and Put had no significant effects on the GA levels in the grains compared with the control group. During the grain filling stage, there was no significant difference in the GA content in the grains among the S1, S2, P1, and CK treatments.

## Discussion

### PA effects on the grain filling of wheat

Grain filling determines the grain weight and represents an important agronomic trait for wheat production. PA was thought to be involved in the regulation of grain filling. During the early and middle grain filling stages of rice, the Spd and Spm contents in superior grains were significantly higher than that in inferior grains; however, the Put content of the superior grains was lower than that of the inferior grains [10]. The grain filling rate and grain weight of rice were positively and significantly correlated with the Spd and Spm contents and the Spd/Put and Spm/Put ratios in the grains, but the grain filling rate and weight were negatively correlated with the Put content of the grains [11]. In the present study, the external application of Spd and Spm significantly increased the grain filling rate and weight of wheat; however, there were no significant differences in these measurements between the P1 and CK treatments (Fig. 1, Table 1). The regression analysis indicated that the endogenous Spm and Spd contents of the grains were positively and very significantly correlated with the maximum grain weight and the maximum and mean grain-filling rates (Table 3). In contrast, the endogenous Put content of the grains was not significantly correlated with the maximum grain weight and the maximum and mean grain-filling rates. This finding indicated that Spm and Spd promote grain filling but that Put has no significant effects on the grain filling rate and weight of wheat. These results are similar to those found in the previous study on rice.

**Table 3:** Correlation coefficients of PA contents in wheat grains with the maximum grain filling rate (G_max_), mean grain filling rate (G_mean_), and maximum grain weight (W_max_) of winter wheat.

	G_max_	G_mean_	W_max_
Spd	0.936**	0.932**	0.940**
Spm	0.933**	0.937**	0.942**
Put	−0.017	0.118	0.292
Spd/Put	0.775*	0.786*	0.768*
Spm/Put	0.775*	0.795*	0.778*
(Spm+Spd)/Put	0.797*	0.711*	0.719*

*Significant at the 0.05 probability level (n = 8).

**Significant at the 0.01 probability level (n = 8).

### Relationship between hormonal changes and grain filling in wheat

CTK reportedly plays an important role in regulating grain filling [27], [28]. In rice, wheat, maize, and barley (*Hordeum vulgare* L.), a higher CTK content was generally observed in the endosperm of the grains, and CTKs were thought to be involved in cell division during the early phase of seed development [12], [13], [14], [15], [16]. In addition to CTKs, IAA plays an important role in regulating the grain filling process [1], [18], [29]. The present study indicated that the Z+ZR contents of the grains were positively and significantly correlated with the maximum grain weight and with the maximum and mean grain-filling rates (Table 4). This finding indicates that Z+ZR is involved in the regulation of wheat grain filling. In addition, the changes in the IAA and Z+ZR contents in the grains appeared to have a very similar pattern. The Z+ZR and IAA contents in grains transiently increased at the early grain filling stage and then decreased, and these two hormones all reached a maximum at 15 days after anthesis. We also observed that the maximum IAA and Z+ZR contents appeared immediately before the maximum grain-filling rate of the two cultivars. Previous studies suggested that CTK [18] and IAA [30] notably regulate endosperm cell division in the developing grains. A high IAA content in the grain could create an “attractive power,” leading to increased CTK contents in the grains [31], [32]. These results suggested that IAA and Z+ZR may regulate wheat grain filling at the early filling stage, most likely by manipulating the division of endosperm cells and thereby creating the sink strength.

**Table 4:** Correlation coefficients of hormone contents in wheat grains with the PA in grains and the maximum grain filling rate (G_max_), mean grain filling rate (G_mean_), and maximum grain weight (W_max_) of winter wheat.

	G_max_	G_mean_	W_max_	Spd	Spm	Put
IAA	−0.056	−0.023	−0.169	−0.164	−0.254	0.148
ABA	0.153	0.187	0.168	0.024	0.064	0.587
Z+ZR	0.708*	0.717*	0.709*	0.491	0.517	−0.485
ETH	−0.820*	−0.745*	−0.759*	−0.730*	−0.740*	0.758*
GAs	0.670	0.667	0.583	0.582	0.581	0.517
ABA/ETH	0.924**	0.814*	0.717*			
ABA/(Z+ZR)	−0.549	−0.482	−0.305			
(Z+ZR)/ETH	0.866**	0.751*	0.721*			

*Significant at the 0.05 probability level (n = 8).

**Significant at the 0.01 probability level (n = 8).

In addition to Z+ZR and IAA, ABA and ETH play important roles in regulating grain filling. Yang et al. [17] suggested that higher ABA concentrations and lower ETH concentrations were found in superior grains compared with inferior grains of wheat and that an increase in the ratio of ABA/ETH promoted the grain filling rate. In the present study, we found a similar result. Our regression analysis indicated that the ETH contents in the grains were negatively and significantly correlated with the maximum grain weight and with the maximum and mean grain-filling rates and that the ratios of Z+ZR/ETH and ABA/ETH were positively and significantly correlated with the maximum grain weight and with the maximum and mean grain-filling rates (Table 4). These results suggested not only that grain filling may be regulated by the absolute level of any individual hormone but also that the balance of hormones may play a more important role in regulating the grain filling of wheat.

There are some reports that GAs are also involved in regulating grain development. A higher GA content was found in the rice grains immediately before and at anthesis [19], [33]. However, the result of the present study showed that the GA content of the grains was not significantly correlated with the maximum grain weight or with the maximum and mean grain-filling rates. In addition, no treatment had significant effects on the GA content in the wheat grains. These results suggested that GAs may not be a major factor in regulating wheat grain filling.

### Relationships between PA and hormones in regulating grain filling

PA reportedly interacts in some way with hormones to regulate the growth and development of plants [6], [34]. PA and ETH were proposed to share the same S-adenosylmethionine biosynthetic precursor, and increasing PA biosynthesis notably affected the ETH synthesis rates [9], [35]. Fuhrer et al. [20] reported that exogenous PAs could repress ETH synthesis in oat leaves. Yang et al. [10] indicated that external applications of Spd or Spm decreased the ETH level in rice panicles and that MGBG applications showed the opposite result. In the present study, we found that different external PAs had different effects on the ETH evolution in wheat grains. Externally applied Spd and Spm significantly decreased the ETH evolution in grains, whereas externally applied Put had the opposite result. In addition, our regression analysis showed that there was a positive and significant correlation between the endogenous ETH and Put contents in the wheat grains but that the endogenous Spm and Spd in wheat grains were negatively and significantly correlated with the ETH content in the grains. These findings indicate that Spd and Spm may inhibit ETH synthesis to promote wheat grain filling; however, Put was unable to significantly promote grain filling because it increased the ETH content in the grains.

Yang et al. [10] indicated that external Spd and Spm significantly increased the Z+ZR levels in inferior rice grains, whereas methylglyoxal-bis (guanylhydrazone) (MGBG), which inhibits the biosynthesis of Spd and Spm, significantly reduced the Z+ZR levels in inferior rice grains. The present study showed that external Spd and Spm significantly increased the Z+ZR contents in wheat grains, whereas external Put had no significant effect on the Z+ZR contents in the grains. These findings indicate that the promoting effects of Spm and Spd were related to the endogenous Z+ZR levels. Additionally, all the PAs, Spm, Spd, and Put significantly increased the ABA content in the grains, and the ABA content after the P1 treatment was significantly higher than that after the S1 and S2 treatments. Steiner et al. [36] indicated that PA increased the endogenous ABA levels. Yang et al. [17] found that the moderately soil-dried treatment (MD) significantly increased the ABA content in rice grains compared with the well-watered treatment (WW) and thereby promoted rice grain filling. However, the severe soil-dried treatment (SD) had the highest ABA content in rice grains compared with the MD and WW treatments, but the grain filling rate and weight of the SD were significantly lower than those of the MD and WW treatments. These results indicated that the moderately increased ABA content promoted grain filling but that an excessively high ABA level may inhibit grain filling. Based on this finding, we suggested that the external Spd and Spm application may increase the ABA content in the grains to promote wheat grain filling and that the inhibitory effect of external Put on grain filling in wheat was related to the excessively high ABA levels in the P1 treatment. These results suggested that PA may regulate wheat grain filling by interacting in some way with hormones and that the PA may regulate the balance of hormones rather than the absolute level of any individual hormone to regulate wheat grain filling. Further studies on the interactions between PA and hormones in the regulation of grain weight of wheat are necessary. For example, the interaction between PA and ETH on the regulation of grain weight should be studied. PA and ETH share the same SAM biosynthetic precursor. However, the relationship between the approach of SAM to PA and the approach of SAM to ETH is unclear. The gene expression pattern and the changes in enzyme activity that regulated the SAM to PA and the SAM to ETH approaches were also unclear. Thus, additional research is needed to investigate the mechanism of grain filling in wheat.

## Conclusions

Exogenous applications of Spd and Spm significantly increased the grain filling rate and the grain weight of wheat, and exogenous Put had no significant effect on the grain filling rate and grain weight. Exogenous Spd and Spm significantly increased the endogenous Spd, Spm, Z+ZR, ABA, and IAA contents and significantly decreased the ETH content in wheat grains. The endogenous Spd, Spm, and Z+ZR contents were positively and significantly correlated with the grain filling rate and weight of wheat, and the endogenous ETH content was negatively and significantly correlated with the grain filling rate and weight of wheat. Based on these results, we concluded that PAs were involved in the balance of hormones needed to regulate wheat grain filling.
